# Development of a modular, biocompatible thiolated gelatin microparticle platform for drug delivery and tissue engineering applications

**DOI:** 10.1093/rb/rbab012

**Published:** 2021-02-28

**Authors:** Hannah A Pearce, Yu Seon Kim, Emma Watson, Kiana Bahrami, Mollie M Smoak, Emily Y Jiang, Michael Elder, Tate Shannon, Antonios G Mikos

**Affiliations:** Department of Bioengineering, Rice University, 6500 Main Street, Houston, TX 77030, USA

**Keywords:** gelatin microparticles, thiolated gelatin microparticles, click chemistry, cell delivery, drug delivery, tissue engineering, biomaterials

## Abstract

The field of biomaterials has advanced significantly in the past decade. With the growing need for high-throughput manufacturing and screening, the need for modular materials that enable streamlined fabrication and analysis of tissue engineering and drug delivery schema has emerged. Microparticles are a powerful platform that have demonstrated promise in enabling these technologies without the need to modify a bulk scaffold. This building block paradigm of using microparticles within larger scaffolds to control cell ratios, growth factors and drug release holds promise. Gelatin microparticles (GMPs) are a well-established platform for cell, drug and growth factor delivery. One of the challenges in using GMPs though is the limited ability to modify the gelatin post-fabrication. In the present work, we hypothesized that by thiolating gelatin before microparticle formation, a versatile platform would be created that preserves the cytocompatibility of gelatin, while enabling post-fabrication modification. The thiols were not found to significantly impact the physicochemical properties of the microparticles. Moreover, the thiolated GMPs were demonstrated to be a biocompatible and robust platform for mesenchymal stem cell attachment. Additionally, the thiolated particles were able to be covalently modified with a maleimide-bearing fluorescent dye and a peptide, demonstrating their promise as a modular platform for tissue engineering and drug delivery applications.

## Introduction

Microparticles are a versatile and powerful platform in biomaterials and drug delivery applications. Microparticles serve as delivery vehicles for cells [1–3], as sites for growth factor or drug delivery [4–8] and as porogens [6, 9, 10] and they can also be utilized as hydrogels or tissue engineering scaffolding systems in their own right without the need for a larger bulk carrier [3, 11–13]. The utilization of microparticles, particularly biocompatible gelatin microparticles (GMPs) as an adherent substrate for cells, has widely been reported to increase cell viability when encapsulated within hydrogels [2, 9]. This system has been demonstrated to be particularly beneficial for adherent cell types such as mesenchymal stem cells (MSCs) and has been harnessed to create robust bone and cartilage tissue engineering systems [9, 14]. Moreover, microparticles have long served as depots for drug or growth factor delivery as a mechanism to control release within tissue engineering scaffolds [6, 15–18]. Bulk encapsulation of drugs within biomaterial scaffolds often results in burst release. The incorporation of drugs within microparticles such as poly(lactic-co-glycolic acid) microparticles or GMPs, though, extends the release profile. This strategy helps maintain drug or factor levels within appropriate dosages for longer periods of time to improve therapeutic outcomes. Furthermore, microparticles have been incorporated into hydrophobic or ceramic scaffolds as a porogen to improve mass and nutrient transfer [10, 19, 20]. Polymeric microparticles can be incorporated into these hydrophobic scaffolds with or without loaded factors to generate porosity which is essential for vascularization, nutrient diffusion and cell infiltration. Lastly, microparticles have served as biomaterial scaffolds without the need for a bulk carrier. Microparticles can be used as a substrate upon which condensates are created to promote cartilage and bone regeneration [12, 13]. Hydrogel microparticles have also been fabricated and then crosslinked together in the presence of cells to create purely microparticle-based bulk hydrogels [3, 11, 21, 22].

Physically and chemically, microparticles have the capability of being highly modular [4, 23, 24]. Traditional biomaterials-based tissue engineering approaches have relied on modifying bulk scaffold properties and studying the relationship between scaffold physicochemical properties and cell behavior. The utilization of microparticles in tissue engineering, though, enables this study to be streamlined [24, 25]. Instead of changing bulk scaffold properties to study the impact of the environment on cells, the physical and chemical environment can be transformed by the addition of varied microparticles in controlled ratios. This strategy enables high-throughput analysis of how the environment impacts cells and tissue formation [13, 26, 27]. The strength of a modular microparticle platform has been emphasized in the development and utilization of 3D-printing to create biomaterial tissue engineering systems [28, 29]. Microparticles can easily be incorporated into biomaterial inks and when coupled with the spatial design allowed through printing, have enabled the creation of highly controlled structures built on modular microparticle building blocks.

This building block, modular paradigm for microparticles has motivated the use of highly specific and functional chemistries within the microparticles themselves. Some of the most robust and functional chemistries for creating modular schema are click chemistry-based systems [3, 4, 11]. The field of click chemistry has been well documented and has yielded the discovery of highly specific, biocompatible and rapid reactions that can be used in biomaterials-based systems that include the thiol-ene reaction, Michael Addition reactions, furan-maleimide reactions and Diels Alder reactions [21, 30–36]. One of the more versatile chemical functional groups utilized within many click reactions is the thiol. Thiols are capable of participating in the thiol-maleimide Michael Addition reaction and the thiol-ene click reaction [3, 21, 37, 38]. Furthermore, thiols can participate in disulfide bond formation which is among the most important biological bonds in stabilizing protein structures. Reaction conditions such as pH, temperature, levels of oxygen and the concentration of available co-reactants can be used to drive and control thiol-based reactions.

As a material, gelatin has remained one of the most biocompatible tissue engineering materials given its biological origins [1, 39]. Gelatin is derived from denatured collagen and depending on the denaturation process used, can possess a majority of pendant carboxylic acid groups (gelatin type A) or free amine groups (gelatin type B). GMPs of types A and B have been well documented as biocompatible cell, drug and growth factor delivery vehicles [2, 5, 6, 40]. Even though gelatin is a large biological molecule, the microparticle crosslinking can be controlled to determine swelling behavior, as well as degradation and release profiles. Chemical modification of gelatin can be performed as well and has yielded a variety of modified gelatins including methacrylated gelatin and thiolated gelatin [1, 39–41].

To create a modular microparticle building block, the ability to chemically modify the microparticles independent of their crosslinking chemistry is vital. To this end, a robust and highly modular thiolated GMP platform was developed. This work sought to build on the well-established foundation of GMPs and demonstrate the capability of adding post-fabrication modification functionality through the addition of thiol groups. The physicochemical properties of microparticles fabricated with thiol-modified and unmodified gelatin was assessed. The modular platform was also examined to determine its capability of serving as a cell or factor delivery vehicle for use in future drug delivery or tissue engineering applications such as osteochondral tissue engineering, wound healing and more.

## Materials and methods

### Thiolated gelatin

The thiolated gelatin was synthesized based on a previously published protocol [40]. Gelatin type B (Nitta Gelatin, Inc. Naniwa-ku, Osaka, Japan) was dissolved in MilliQ water at 37°C at 1% w/v. Traut’s reagent (Thermofisher Scientific, Waltham, MA) was added to the dissolved gelatin at 20 mg or 80 mg Traut’s reagent per 1 g of gelatin (Gel-SH 20 and 80) and allowed to stir overnight while protected from light at 37°C. The thiolated gelatin was then collected and purified via dialysis in 5 and 1 mM HCl (ThermoFisher Scientific, Waltham, MA) in MilliQ water for 24 h each at 37°C. Following dialysis, the solutions were flash frozen and freeze-dried to isolate the thiolated gelatin groups. Once dry, all thiolated gelatin was stored at –20°C.

### Microparticle formation

The thiolated GMPs were formed (GMP-SH 20 and 80) using a modified protocol previously established in the laboratory [2, 5, 6]. GMPs were formed alongside all GMP-SH groups as an internal control. Briefly, 2.5 g thiolated or non-thiolated gelatin was dissolved in 22.5 ml MilliQ water over low heat (40–50°C) at 10 wt% to dissolve the thiolated or non-thiolated gelatin without promoting disulfide bond formation through excessive heat exposure. The pH of the thiolated gelatin solution was adjusted to match that of the non-thiolated gelatin solution (pH 5.4) by adding NaOH (MilliporeSigma, St. Louis, MO).

Once fully dissolved, the mixture was added dropwise to a 3-neck flask connected to an overhead stirring solution of 125 ml olive oil (MilliporeSigma, St. Louis, MO) and 0.5 wt% SPAN80 (MilliporeSigma, St. Louis, MO) set at 400 rpm. Once addition of the gelatin solution was complete, the mixture was stirred for 30 min on ice. 50 ml chilled acetone (ThermoFisher Scientific, Waltham, MA) was then added to the stirring suspension and the mixture was left to stir for 1 h. The microparticles were collected by pouring the solution over size 40 filter paper (Whatman, Maidstone, UK), washed several times with chilled acetone to remove any remaining oil phase and stored at –20°C while the crosslinking stage was assembled. Several mg of uncrosslinked microparticles were saved at this phase for crosslinking density analysis.

The crosslinking setup consisted of 250 ml chilled MilliQ water containing 0.1% Tween80 (MilliporeSigma, St. Louis, MO). This mixture was poured into a 3-neck flask and stirred via overhead stirring at 400 rpm while surrounded by ice. Glutaraldehyde solution (MilliporeSigma, St. Louis, MO) was added to the crosslinking phase at 40 mM final concentration. While stirring, the microparticles were added slowly to the crosslinking phase. The suspension was left to stir on ice for 20 h. Following the crosslinking reaction, glycine (MilliporeSigma, St. Louis, MO) was added to the mixture at 25 mM final concentration to react for 1 h to quench any unreacted aldehyde groups. The final filtration and purification was performed by washing the particles over size 40 filter paper with chilled water:acetone mixtures (100:0, 75:25, 50:50, 25:75, 0:100). The particles were collected and frozen at –20°C before freeze-drying.

Particles between 50 µm and 100 µm while dry were used for all studies noted below and were isolated via sieving. Microparticle size following sieving was determined via image-based analysis on particles swollen in phosphate buffered saline (PBS). Briefly, particle diameter measurements were completed on the first 10 microparticles that crossed the median of each 10× magnification image. Average microparticle sizes are reported ± standard deviation. All particles were stored dry under N_2_ gas at –20°C and allowed to reach room temperature before use.

### Microparticle physicochemical characterization

Microparticle physicochemical properties such as swelling behavior, crosslinking density, degradation behavior and thiol content before and after crosslinking were all examined based on existing protocols [2, 5, 6, 42].

*Thiol Content Before and After Microparticle Crosslinking—*The degree of thiolation of Gel-SH and GMP-SH groups was performed via a modified Ellman’s assay reported previously [42]. 30 mg of gelatin, Gel-SH, GMP and GMP-SH groups were all dissolved or swollen in 0.25 ml 0.1 N acetic acid solution (Thermofisher Scientific, Waltham, MA). 0.25 ml of 6 N NaOH (MilliporeSigma, St. Louis, MO) was then added and allowed to incubate on a shaker table at 37°C for at least 1 h to swell and degrade the microparticles and reduce disulfides. 0.5 ml of 6 N H_3_PO_4_ (MilliporeSigma, St. Louis, MO) with 2 mM EDTA (MilliporeSigma, St. Louis, MO) was then added to neutralize the solution before adding 0.1 ml Ellman’s reagent [5,5-dithio-bis-(2-nitrobenzoic acid)] (Thermofisher Scientific, Waltham, MA) dissolved at a 1 mg/ml concentration in 0.02 M sodium acetate solution (Thermofisher Scientific, Waltham, MA) and incubated 15 min. The absorbance was then measured at 412 nm using a cysteine standard treated in the same manner with known concentrations of 0–1.5 mM cysteine (MilliporeSigma, St. Louis, MO). Due to the imines formed from the glutaraldehyde-amine crosslinking known to absorb at 412 nm, all groups had an internal control of their respective samples prepared in the same manner without the Ellman’s reagent solution. This enabled the absorbance of the dissolved and partially degraded groups without the active agent to be subtracted as a blank. The molar content of gelatin was determined by mass and the molecular weight provided by the distributor of approximately 170 kDa. The molar content of the microparticles after crosslinking was determined by mass as well. Since the mass ratio of gelatin in the microparticles far surpassed that of the short chain glutaraldehyde, the mass contribution of glutaraldehyde to the microparticle weight was assumed to be negligible. An *n* = 3 replicates per group were used.

*Swelling Behavior—*The swelling behavior of the microparticle groups was assessed in PBS (MilliporeSigma, St. Louis, MO) at 37°C. All GMP-SH groups were assessed compared to a GMP control. 5 mg of microparticles were placed in dried and weighed centrifuge tubes (Thermofisher Scientific, Waltham, MA) fitted with 5 µm pore size filters and swollen overnight at 37°C in 0.5 ml PBS. An *n* = 5 replicates were used for each group. Following swelling, the centrifuge tubes were spun down at 2000 rpm for 3 min and the PBS removed before weighing again to determine the microparticle wet weight. The swelling ratio was determined using the equation below where *W*_S_ is the swollen microparticle weight and *W*_D_ is the dry microparticle weight.

$$\mathrm{Swelling}\;\mathrm{Ratio}=\;\frac{W_{S}-W_{D}}{W_{D}}$$

*Crosslinking Density—*The crosslinking density of the microparticle groups was examined via 2,4,6-trinitrobenzene sulfonic acid (TNBSA) assay (Thermofisher Scientific, Waltham, MA) and percent crosslinking was determined based on a method reported previously [43]. Briefly, 3 mg of uncrosslinked or crosslinked microparticles were dissolved or swollen in 5 ml of 0.1 M sodium bicarbonate (MilliporeSigma, St. Louis, MO) buffer for 1 h at 37°C. 0.5 ml of that solution was then taken and combined with 0.25 ml 0.01 w/v% TNBSA solution in 0.1 M sodium bicarbonate buffer. This mixture was left to react 2 h at 37°C before the addition of 0.125 ml 1 N HCl. The absorbance was measured for all groups using known weights of gelatin as a standard at 335 nm and all samples were normalized by weight with an *n* = 3 replicates used per group. The crosslinking density was assessed as percent lysine loss and calculated with the equation below where *A*_C_ is the absorbance of the crosslinked microparticle groups and *A*_U_ is the absorbance of the uncrosslinked microparticle groups.

$$\mathrm{Percent}\;(\%)\;\mathrm{Lysine}\;\mathrm{Loss}=\left(1-\frac{A_{C}}{A_{U}}\right)\times 100$$

*Degradation Behavior—*Microparticle degradation was assessed in sterile filtered PBS with and without 800 ng/ml collagenase type I (MilliporeSigma, St. Louis, MO) over a 40-day period at 37°C with sampling and solution changes performed at day 1 and then biweekly for the remainder of the study. Sterile filtered PBS was utilized, and solution changes were performed in a biosafety cabinet to prevent contamination. 10 mg of dried particles were placed in 5 ml of degradation medium in centrifuge tubes to shake at 37°C with an *n* = 4 replicates per group. For each solution change, 4 ml of the medium was removed and stored for analysis with fresh medium replacing it. A MicroBCA assay (ThermoFisher Scientific, Waltham, MA) of the supernatant was performed at each time point to measure the mass loss of the GMP and GMP-SH groups as reported previously [2]. The total amounts degraded were reported as percent mass loss measured from the initial mass of microparticles in each group.

### Leachables biocompatibility assessment

The biocompatibility of the microparticle leachables was established according to well-documented leachables biocompatibility assays [44]. L929 fibroblasts were grown to confluency in T75 flasks and plated in 96-well plates at 10 000 cells/well before passage 12 and allowed to grow 24–48 h to reach confluency. The cells were grown in general culture medium containing low glucose (LG)–DMEM (ThermoFisher Scientific, Waltham, MA), 10% FBS (Gemini Bio-Products, Sacramento, CA) and 1% penicillin/streptomycin (ThermoFisher Scientific, Waltham, MA). All microparticle groups were sterilized via EO gas exposure (Andersen Products, Haw River, NC) and then incubated in serum-free general culture medium at 10 mg/ml concentration for 24 h. Medium containing microparticle leachables was then sterile filtered and applied to confluent L929 fibroblasts at 1, 10 and 100x dilutions with cells exposed to serum free medium used as a control. An *n* = 3 replicates per group was utilized. The metabolic activity of the cells was assessed at 2 h and 24 h following leachables exposure via WST1 assay (MilliporeSigma, St. Louis, MO) and the DNA content of the wells was assessed via Picogreen (ThermoFisher Scientific, Waltham, MA) assay following 100 µg/ml proteinase K digestion (ThermoFisher Scientific, Waltham, MA) and three freeze/thaw cycles to release dsDNA.

### MSC isolation and culture

MSCs were harvested from the tibiae of 6-month-old male New Zealand White rabbits according to well-established protocols [9, 45, 46] and in compliance with the Institutional Animal Care and Use Committee of Rice University. The MSCs were plated upon harvest and cultured in growth medium [LG-DMEM (ThermoFisher Scientific, Waltham, MA), 1% Antibiotic-Antimycotic (ThermoFisher Scientific, Waltham, MA), 20% FBS (Gemini Bio-Products, Sacramento, CA)] until 80% confluent before freezing down and storing under liquid nitrogen vapor. Before use, the MSCs were thawed and plated at passage 1 and grown with medium containing LG-DMEM, 1% Antibiotic-Antimycotic and 10% FBS. All MSCs were used at passage 3. The MSCs were lifted using TryplE (ThermoFisher Scientific, Waltham, MA) and counted using a hemocytometer before use.

### MSC adhesion to thiolated and non-thiolated GMPs

MSCs were adhered to the surface of GMP and GMP-SH groups following a modified version of a previously established protocol [47]. Briefly, the GMP and GMP-SH groups were sterilized via EO gas exposure. MSCs were incubated in a rotational culture setup at 300 000 cells/ml with 1 mg/ml microparticle group (GMP, GMP-SH 20, or 80) at 10 RPM. The cell and microparticle mixtures were left to adhere overnight and collected using a 40 µm cell strainer. The isolated cell and microparticle aggregates were washed with PBS and either stained via Hoechst-33342 (ThermoFisher Scientific, Waltham, MA) staining to visualize the MSCs on the microparticle surface, returned to rotational culture to determine metabolic activity of the MSC and microparticle aggregates at 2 h and 24 h, or isolated and frozen for Picogreen analysis.

*Percent (%) MSC Adherence—*MSC adherence to the microparticle groups was determined from three independent adherence trials via Picogreen assay (ThermoFisher Scientific, Waltham, MA). Following overnight adherence culture, the MSC and microparticle aggregates were collected, washed and suspended in PBS. The MSC and microparticle aggregates and the wash solutions were separately subjected to three freeze/thaw cycles to release dsDNA into solution. The percent MSC adherence was determined as fractional DNA content of the lysed MSC and microparticle aggregate groups (DNA_MP_) over the summed DNA content of the aggregates (DNA_MP_) and wash solution (DNA_Wash_) using the following equation:

$$\mathrm{Percent}\;\left(\%\right)\;\mathrm{MSC}\;\mathrm{Adherence}=\;\frac{{\mathrm{DNA}}_{\mathrm{MP}}}{{\mathrm{DNA}}_{\mathrm{MP}}+{\mathrm{DNA}}_{\mathrm{Wash}}}$$

Metabolic activity of the adhered MSCs was also determined by WST1 assay with *n* = 3 replicates per group and the metabolic activity of the MSCs adhered to GMP-SH 20 and 80 groups was compared to the activity of MSCs adhered to GMP controls. All WST1 data was normalized to adhered MSC dsDNA content determined via Picogreen for each group.

### Modification of the thiolated particles with Alexa Fluor-Maleimide 488

To demonstrate the modularity and ability to covalently modify the thiolated microparticle groups post-fabrication, the GMP and GMP-SH groups were reacted first with an Alexa-Fluor Maleimide (AF-Mal) 488 (Fluoroprobes, Scottsdale, AZ) to allow thiol-maleimide chemistry to covalently link the dye to the microparticles. In addition to demonstrating the feasibility of utilizing thiol-maleimide chemistry to covalently load the microparticles, the dye tethering also served as a proof-of-concept to demonstrate the particles’ ability to serve as a small molecule carrier. Briefly, the particles were swollen in deoxygenated TRIS base buffer (pH 7.5) (MilliporeSigma, St. Louis, MO) and reacted with 10 molar equivalents of Tris(2-carboxyethyl)phosphine hydrochloride (TCEP) (MilliporeSigma, St. Louis, MO) to reduce disulfides. The microparticle groups were then reacted with a 5-molar excess of AF-Mal 488 in deoxygenated buffer and allowed to react overnight before adding an excess of dithiothreitol (MilliporeSigma, St. Louis, MO) to react with any remaining dye. The microparticles were then washed to remove any unreacted dye. The fluorescence of the particles was quantified via microplate assay at 485 nm excitation, 525 nm emission with *n* = 4 replicates per group. The relative fluorescence units (RFU) of each group was quantified using the following equation (ThermoFisher Scientific, Waltham, MA) for quantifying fluorescent covalent modification where *A*_x_ denotes absorbance of the sample and *ε* is the extinction coefficient of the dye.

$$\mathrm{Degree}\;\mathrm{of}\;\mathrm{Labeling}=\;\frac{A_{x}}{\varepsilon }\;\times \;\frac{MW\;\mathrm{of}\;\mathrm{protein}}{\frac{mg}{mL}of\;\mathrm{protein}}\;=\;\frac{\mathrm{mol}\;\mathrm{dye}}{\mathrm{mol}\;\mathrm{protein}}$$

### Modification of the thiolated microparticles with an N-Cadherin mimic peptide

To demonstrate the potential of the microparticles as a delivery vehicle for small biomolecules or drugs, an N-cadherin mimic peptide bearing a maleimide group (‘Mal-GDGGHAVDI’) was synthesized and covalently tethered to the thiolated microparticles. The peptide was synthesized as reported previously using solid phase synthesis and FMOC-based chemistry [33, 48, 49] using a rink amide MBHA resin. Following peptide synthesis and cleavage from the resin, the product was collected via ether precipitation before dialyzing against MilliQ water 24 h to purify. The peptide sequence was confirmed via MALDI-TOF and ^1^H-NMR analysis, the spectra for which are provided Supplementary Figs. S4 and S5, respectively.

To modify the microparticles with the peptide, the particles were swollen in deoxygenated TRIS base buffer (pH 7.5) (MilliporeSigma, St. Louis, MO) and reacted with 10 molar equivalents of Tris(2-carboxyethyl)phosphine hydrochloride (TCEP) (MilliporeSigma, St. Louis, MO) to reduce disulfides. The microparticle groups were then reacted with a 25-molar excess of peptide in deoxygenated buffer and allowed to react overnight before washing to remove any unreacted peptide.

The degree of peptide modification on the microparticle groups was determined via Ellman’s assay as reported above. The percent thiol modification was calculated according to the equation below by subtracting the absorbance of the peptide-modified groups (*A*_peptide_) from the absorbance of the unmodified groups (*A*_unmodified_) and dividing by the absorbance of the unmodified group (*A*_unmodified_).

$$\mathrm{Percent}\;\left(\%\right)\;\mathrm{Thiol}\;\mathrm{Modification}=\;\frac{A_{\mathrm{unmodified}}-\;A_{\mathrm{peptide}}}{A_{\mathrm{unmodified}}}$$

To report the molar equivalents of peptide loaded into the thiolated particles, the percent modification calculated above was multiplied by the known thiol molar content of each GMP-SH group.

### Statistics

Biochemical assays were analysed using one-way analysis of variance followed by Tukey’s Honest Significant Difference post-hoc test (*P* < 0.05) or Sidak’s multiple comparisons test when comparing different numbers of replicates (*P* < 0.05). All statistical tests were conducted and obtained using JMP Pro 14 (SAS Institute, Cary, NC) and GraphPad Prism (GraphPad Software, La Jolla, CA). Data points are displayed as mean ± standard deviation, unless otherwise noted.

## Results and discussion

### Physicochemical characterization of thiolated microparticles

**Figure 1.**
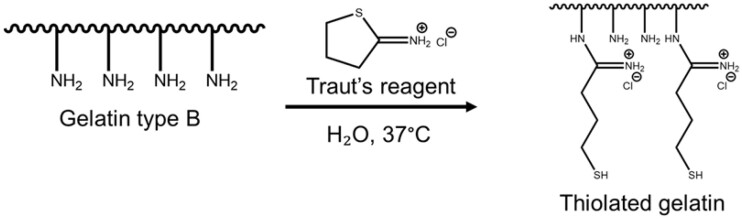
Schematic of gelatin thiolation. Gelatin type B was reacted with 20 or 80 mg Traut’s reagent per 1 g gelatin to form Gel-SH 20 and 80

The ability to covalently modify GMPs post-fabrication enables their use as a modular building block in a variety of tissue engineering applications such as bone and cartilage tissue engineering, wound healing applications and more. For this novel work, thiols were chosen as a moiety to add to the gelatin to allow covalent modification after microparticle formation. Unlike previous reports of thiolated gelatin nanoparticles [40], this utilization of GMP-SHs as a delivery platform requires crosslinking chemistry independent of the thiols so they are available for subsequent use after particle formation. Thiols offer a variety of biocompatible and highly specific click and non-click reactions with which to operate [3, 21, 30, 40]. To achieve this capability of post-fabrication modification, gelatin type B was reacted with 20 mg or 80 mg Traut’s reagent per 1 g gelatin to represent a high and low thiol-content gelatin (Gel-SH 20 and 80). These concentrations of Traut’s reagent with respect to the gelatin were previously established by others as being a lower and upper bound for the addition of thiols for the purposes of intracellular DNA delivery [40]. A schematic of the thiolation reaction is provided in Fig. 1.

**Figure 2.**
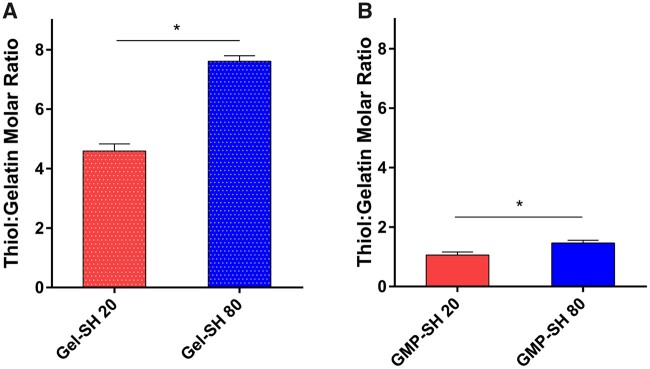
Thiol content for the thiolated gelatin and thiolated GMP groups. (**A**) Thiolated gelatin (Gel-SH 20 and 80) and (**B**) thiolated GMP groups (GMP-SH 20 and 80) were assessed for thiol content via a modified Ellman’s assay using a cysteine standard curve. Data is reported as means ± standard deviation for *n* = 3 replicates per group. *Indicates significant differences between groups (*P* < 0.05)

**Figure 3.**
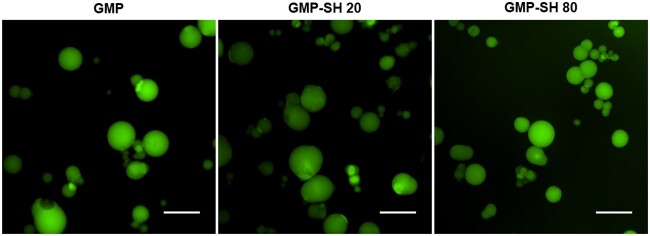
Representative fluorescent images of gelatin and thiolated GMP groups. Fluorescent microscopy images of microparticles swollen in PBS (50–100 µm while dry). Scale bar = 200 µm

The results shown in Fig. 2A demonstrate a dose-dependent response in the thiol content of soluble thiolated gelatin as more Traut’s reagent is used between the Gel-SH 20 and 80 groups with a thiol to gelatin molar ratio of 4.6 ± 0.2 and 7.6 ± 0.2 for Gel-SH 20 and 80, respectively. This significant difference in dose-dependence remains in the GMP-SH groups as seen in Fig. 2B, though reduced from the levels observed in the soluble thiolated gelatin. The thiol to gelatin molar ratio of the GMP-SH 20 and 80 groups were 1.1 ± 0.1 and 1.5 ± 0.1, respectively. We hypothesize that the reduction in thiols observed in the thiolated microparticles compared to the soluble thiolated gelatin is partially due to thiol detection being limited to the thiols on the surface and near the surface of the GMP-SH groups. Ellman’s reagent reacts with free thiols, and the luminescent species that is generated must then diffuse out of the network to be detected. The Ellman’s protocol that was utilized partially degraded the microparticles to aid in better thiol quantitation, but it is possible that some reagent did not penetrate into the network, or that the luminescent species formed by reacting with free thiols was not able to diffuse out of the network in the short 15 min incubation time for detection. Furthermore, it is possible that minimal amounts of thiols are consumed by the glutaraldehyde crosslinker [50]. Thiols can react with aldehydes to form a reversible hemithioacetal bond. This reaction is temporary as it is a transition in the formation of thioacetals [51]. Thioacetal formation, however, requires a dithiol and we hypothesize that since the gelatin backbone is a large macromolecule with respect to the short-chain glutaraldehyde, the proximity of other thiols required to transition from a hemithioacetal to a permanent thioacetal bond are miniscule. Thus, negligible amounts of thiols should be consumed by reacting with the glutaraldehyde. Nevertheless, from the GMP-SH data, it was demonstrated that the two groups bear statistically significant thiol content representing a high and low thiol content microparticle group. As a result, these two groups were carried through the remainder of the study to determine the impact of thiols on the physicochemical properties of the microparticles and their capability of serving as a cell and factor delivery vehicle compared to GMP controls. Microparticle size distribution was determined via image-based analysis of particles swollen in PBS with average particle sizes determined to be 125.2 ± 34.8, 81.4 ± 46.1 and 101.4 ± 40.6 µm for the GMP, GMP-SH 20 and GMP-SH 80 groups, respectively. The size distribution of the microparticles is provided in Supplementary Fig. S1 and demonstrates no significant difference in size between groups. Representative images of the microparticles swollen in PBS containing 0.1% fluorescein can be seen in Fig. 3.

**Figure 4.**
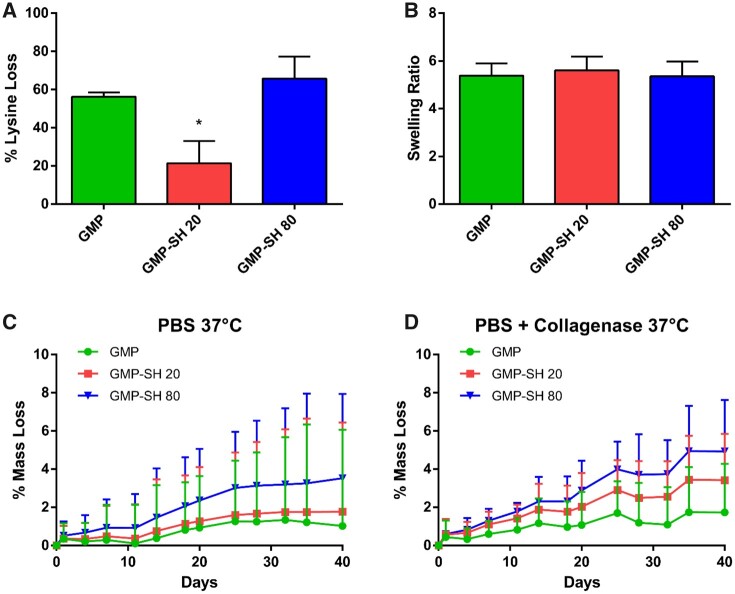
Physicochemical characterization of the thiolated GMP groups. (**A**) Crosslinking density was determined as % lysine loss measured via TNBSA assay. Data is reported as means ± standard deviation for *n* = 3 replicates per group. *Indicates significant difference (*P* < 0.05). (**B**) Swelling ratio of the thiolated particle groups compared to GMP control. Data is reported as means ± standard deviation for *n* = 5 replicates per group. No statistical significance was detected (*P* < 0.05). (**C**) Degradation of the thiolated and control GMP groups in PBS at 37°C. Data is reported as means + standard deviation for *n* = 4 replicates per group. (**D**) Degradation of the thiolated and control GMP groups in collagenase-containing medium at 37°C. Data is reported as means + standard deviation. *N* = 4 replicates per group

The impact of thiols on the swelling, crosslinking and degradation behavior of the microparticles was assessed for both GMP-SH groups compared to a GMP control. The crosslinking density represented as lysine loss as shown in Fig. 4A demonstrated a significantly lower degree of crosslinking for the GMP-SH 20 group compared to GMP-SH 80 and the GMP controls. Interestingly, the presence of thiols and this difference in crosslinking density between the GMP-SH 20 and 80 groups was not found to influence swelling behavior, as shown in Fig. 4B. Similar swelling behavior between the GMP-SH groups and the GMP control demonstrates their versatility for use as additives in a variety of applications such as injectables or extrusion-based 3D printing.

While the presence of thiols was demonstrated to increase the rate of degradation in PBS and accelerated conditions (PBS + collagenase) in a dose-dependent manner as shown in Fig. 4C and D, the mass loss after 40 days was less than 10% for the GMP-SH 80 group, demonstrating the stability of the GMP-SHs even in accelerated conditions designed to mimic the wound-healing environment [6]. The complete letter report indicating significance in the degradation data is provided in Supplementary Fig. S2. In brief, no significant difference between groups was observed in the PBS degradation conditions at each timepoint. However, there was significance observed in the degradation of the GMP-SH 80 groups compared to the GMP controls in the collagenase-containing conditions, particularly at the days 35 and 40 timepoints. Importantly, the GMP-SH 20 group still demonstrated stability in the degradation studies despite the lower crosslinking density, demonstrating its promise as a delivery vehicle in tissue engineering applications. Previously, our group has demonstrated the ability to tune degradation for the purposes of growth factor release by varying the glutaraldehyde crosslinker concentration during microparticle fabrication [6, 17, 52]. A similar schema could be used of reducing the glutaraldehyde concentration from 40 mM to 10 mM in the future if a more accelerated degradation profile is required [6, 15, 17, 52].

**Figure 5.**
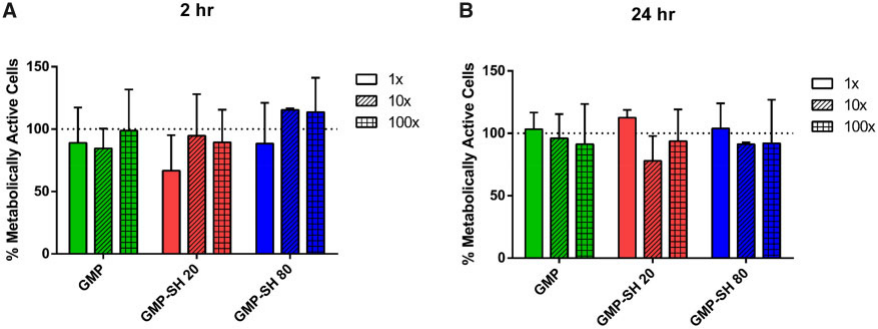
WST1 Metabolic assay of L929 fibroblasts treated with microparticle leachables. Metabolic activity of the cells was assessed via WST1 assay at (**A**) 2 h and (**B**) 24 h following leachables exposure. Data is reported as means ± standard deviation for *n* = 3 replicates per group. No statistical significance was found between groups within the same timepoint (*P* < 0.05)

A leachables cytocompatibility assay was performed for both GMP-SH groups compared to a GMP control. As shown in Fig. 5A and B, the leachables were demonstrated to be cytocompatible for the cells at 2 h and 24 h at all dilutions with no significant difference in metabolic activity observed between cells exposed to GMP soluble leachables and either of the GMP-SH groups. The dashed line at 100% represents the metabolic activity of L929’s exposed to serum-free media controls. The dsDNA content of each experimental group of L929 fibroblasts was also determined via Picogreen assay to ensure comparisons across groups and dilutions were of comparable cell numbers. The data demonstrated no significant difference in dsDNA content across groups and dilutions and is shown in Supplementary Fig. S3.

### Thiolated GMPs as a stem cell carrier

**Figure 6.**
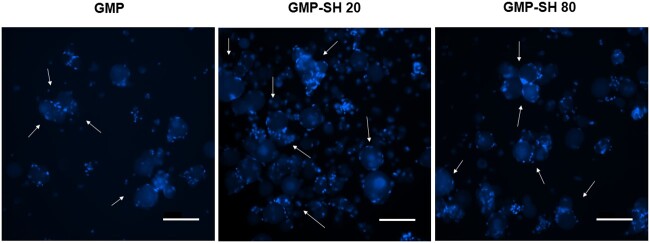
Nuclei staining of MSCs adhered to gelatin and thiolated GMPs. White arrows indicate some of the MSC and microparticle aggregates swollen in PBS and stained with Hoechst-33342. Scale bar = 200 µm

GMPs are a well-characterized cell delivery and cell surface attachment substrate [1, 2, 9]. However, the use of GMP-SHs as a cell delivery platform is unique. To demonstrate the modularity and strength of the GMP-SHs as a cell-delivery platform technology, MSCs were adhered to the microparticle surface. Representative images of the cell and microparticle aggregates can be seen below in Fig. 6. Robust and comparable cell attachment is observed for both GMP-SH groups compared to the GMP control. For some of the groups, the MSCs even appear to be acting as a bridge in the formation of aggregates containing multiple microparticles. Microparticle-based aggregate cultures have been reported to serve as robust osteochondral tissue engineering platforms [12, 13, 53, 54] that allow for the controlled presentation of growth factors or other bioactive factors to adhered cells. GMP-SHs show promise as a platform for building these microparticle-based aggregate culture given their biocompatibility and chemical functionality.

**Figure 7.**
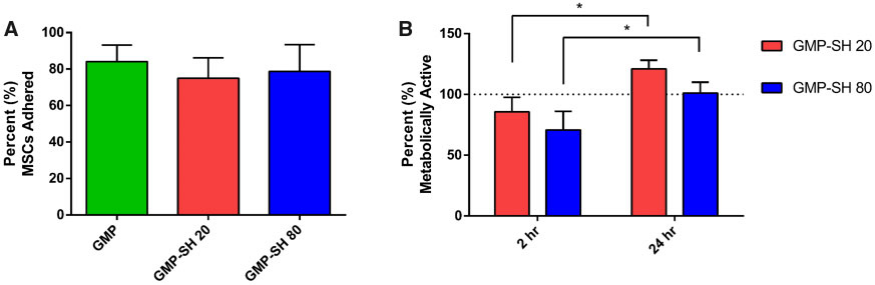
Quantification of MSC adherence to gelatin and thiolated GMPs and adhered MSC metabolic activity. MSC-microparticle aggregates were analysed via picogreen assay to determine (**A**) percent (%) adherence to the microparticles. Data is reported as means ± standard deviation for *n* = 3 replicates per group. No significant difference was detected between groups (*P* < 0.05). (**B**) The aggregates were cultured in rotational culture and the metabolic activity of the adhered MSCs was examined at 2 h and 24 h via WST1 assay. Data is reported as means ± standard deviation for *n* = 3 replicates per group. The dashed line indicates the activity of MSCs adhered to GMP controls. *Indicates significant differences within each group between the activity observed at 2 h compared to 24 h (*P* < 0.05)

To quantify this cell attachment, the percent MSC adherence was calculated and is outlined in Fig. 7A. As shown, the MSC attachment to the GMP-SH 20 and 80 groups was comparable to the attachment observed in the GMP group. To ensure that the MSCs were not only adhered to the microparticle surface, but also viable and active, the MSC-microparticle aggregate groups were cultured in rotational culture for 24 h and their metabolic activity was assessed at 2 h and 24 h, as shown in Fig. 7B. The activity of the MSCs adhered to the GMP-SH groups was assessed as a fractional percentage of the activity observed for MSCs adhered to the GMP control, represented as the dotted line at 100% on the graph. Picogreen dsDNA data was used to normalize metabolic activity for each group. The metabolic activity of the adhered MSCs decreased for the cells adhered to the GMP-SH groups at 2 h with respect to activity on the GMP control. However, in looking at the 24 h metabolic activity data, the MSCs make a complete recovery and return to comparable metabolic activity levels as observed of the MSCs adhered to GMPs. The activity within each microparticle group at 2 h was compared to the activity at 24 h and the MSCs make a statistically significant recovery within each group from the activity observed at 2 h compared to 24 h.

This robust cell attachment and ability to serve as a cell culture substrate demonstrates the strength of the GMP-SH platform. GMPs are a well-known, biocompatible cell-attachment substrate [2, 9, 13] and the ability to match this biocompatibility in GMP-SHs demonstrates the versatility of the thiolated microparticles. Additionally, the interaction of the thiols and the cells attached to the surface is an area of future opportunity in investigating the direct impact the thiols on the particle surface have with the adhered cells, as well as any indirect impacts in the form of bound bioactive factors and their impact on cell behavior.

### Modification of the thiolated particles with small molecules

To create a modular building block platform for varied applications, the ability to covalently modify the GMP-SHs is paramount. This post-fabrication modification enables the tethering of proteins, bioactive molecules, drugs and more to the microparticle surface. GMPs have long served as a delivery vehicle for growth factors, drugs and more but these schemas have largely relied on physical interactions of the gelatin with the bioactive factors of interest [5, 6, 17]. Thiolated gelatin nanoparticles have been formed previously to aid in intracellular delivery of DNA but these nanoparticles were designed to be taken up into the cells and degraded upon exposure to reducing compounds [40]. The development and utilization of GMP-SHs for the delivery of cells and the external presentation of bioactive factors is unique.

**Figure 8.**
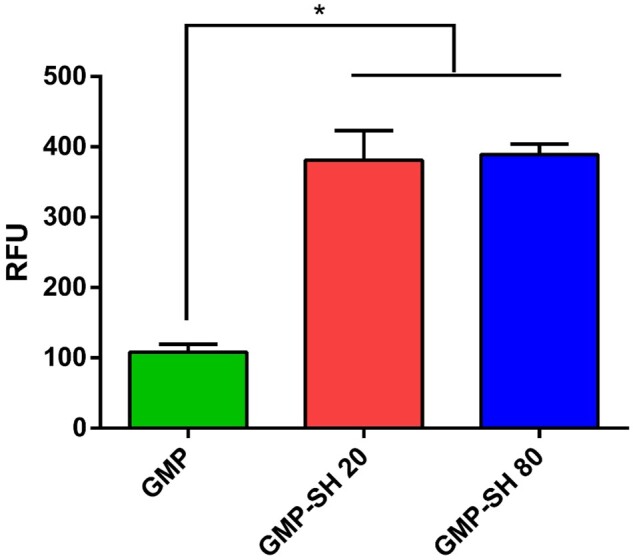
Fluorescence of microparticle groups incubated with Alexa Fluor-Maleimide (AF-Mal) 488. Fluorescence of the microparticles was analysed and normalized to gelatin content. Data is reported as means ± standard deviation for *n* = 3 replicates per group. *Indicates significant differences between groups (*P* < 0.05)

To demonstrate proof of concept of the thiol-maleimide reaction, the GMP-SHs were combined with an Alexa-Fluor maleimide dye. Maleimides react readily with thiols in aqueous conditions at neutral pH [38, 55]. It was hypothesized that the maleimide dye would selectively bind with the thiols on the GMP-SHs and this would correspond to increased fluorescence observed for the GMP-SH groups even after washing the microparticles. Both GMP-SH groups and a GMP control were reacted with the dye and the RFUs normalized by microparticle content within each group are reported in Fig. 8.

As demonstrated by the data, increased fluorescence was observed for both GMP-SH groups compared to the GMP controls, indicating successful covalent modification of the dye for the thiolated particles. Interestingly, the dose-dependent thiol content of the GMP-SH groups did not correspond to dose-dependence in the content of the hydrophobic fluorescent dye within the microparticles. This demonstrates that, at least for this hydrophobic small molecule, the microparticles of the lowest thiol content, GMP-SH 20, could be utilized to maximize loading while minimizing the thiol content in the microparticle carrier.

**Figure 9.**
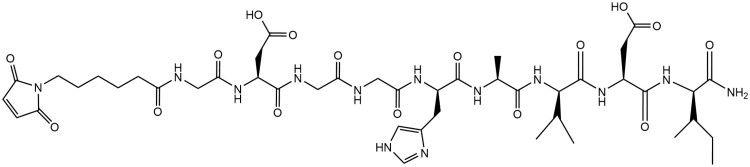
Maleimide-Bearing N-Cadherin mimic peptide. The N-cadherin mimic peptide bearing a C-terminal maleimide group was synthesized via solid phase, FMOC peptide chemistry. The peptide sequence was confirmed via MALDI TOF and ^1^H-NMR

To investigate the capability of loading small biomolecules such as peptides into the thiolated microparticles, an N-cadherin mimic peptide bearing a maleimide was synthesized and covalently loaded into the microparticles. The peptide structure is given below in Fig. 9. The MALDI TOF and ^1^H-NMR spectra confirming the correct sequence are provided in Supplementary Figs. S4 and S5, respectively.

**Figure 10.**
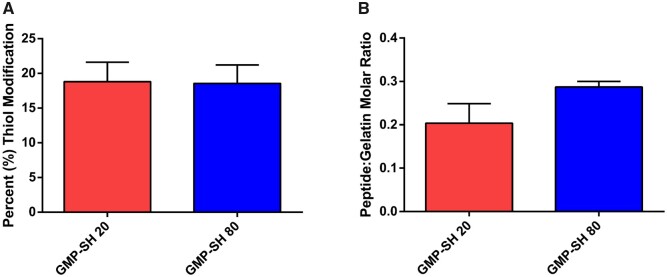
Modification of the thiolated microparticles with an N-Cadherin mimic peptide. The percent thiol modification (**A**) with the maleimide-bearing peptide was determined via Ellman’s assay. Data is reported as means ± standard deviation for *n* = 4 replicates per group. No statistical significance was found between groups (*P* < 0.05). The peptide molar modification ratio (**B**) was then calculated using the known molar content of the GMP-SH 20 and 80 groups. Data is reported as means ± standard deviation for *n* = 3 replicates per group. No statistical significance was found between groups (*P* < 0.05)

The successful loading of the peptide into the thiolated microparticle network was confirmed via Ellman’s assay. Comparable percent thiol modification was observed, as shown in Fig. 10A, demonstrating comparable reaction efficiency of the peptide with the two microparticle groups. Interestingly, dose-dependent loading of the peptide within the GMP-SH 20 and 80 was demonstrated, as shown in Fig. 10B, albeit the difference was not significant (*P* = 0.07). Unlike the AF-Mal 488, the peptide loaded into the microparticles is hydrophilic and can thus more easily diffuse into the microparticle network. The molar content of peptide per microparticle group was calculated with the known thiol molar content of GMP-SH 20 and 80 as presented above in Fig. 2B. Though the differences in loading between the GMP-SH 20 and 80 were not significant, the platform still demonstrates promise as a carrier for dosed loading of bioactive factors and small molecules.

Future work will investigate the ability to control the loading of bioactive factors within the GMP-SH 20 and 80 groups to tune biological effects. Upon loading and presentation to cells, the biological effects of the loaded bioactive factors may mirror the significant difference in thiol content as seen in the Ellman’s assay data in Fig. 2B. In *in vitro* or *in vivo* culture, the load of bioactive factor tethered to the GMP-SH 20 and GMP-SH 80 groups may still bear significantly different influence over cell behavior that a fluorescence-based assay or peptide tethering determined via Ellman’s assay cannot determine.

Future work will also explore the dose-dependence in the loading of larger, thiol-reactive biomolecules such as growth factors. These large molecules will react with the surface thiols on the microparticles and serve as a barrier for diffusion further into the microparticle network. Consequently, these large biomolecules may demonstrate a true dose-dependence in their surface presentation between the GMP-SH 20 and 80 groups.

This capability of covalently loading GMP-SHs holds promise for the selective and controlled delivery of peptides [33], growth factors [15, 56], drugs [16] and more. The thiol-maleimide bond, specifically, is highly stable [32] and offers the capability of long-term presentation of bioactive factors. This is in sharp contrast to the transient and short-term presentation observed in systems reliant on adsorption or other temporary interactions traditionally observed in GMP platforms [5, 6]. The linkage by which these factors are covalently tethered onto the microparticles could also be designed to be cleavable by hydrolysis, pH change, or even matrix metalloproteinase activity [31] to create a stimuli-responsive controlled release system. Thiols are a highly versatile chemical moiety and can thus be harnessed within the GMP-SH platform to deliver factors separately from the crosslinking chemistry. As discussed previously, in addition to the thiol-maleimide reaction, thiol-ene chemistry and disulfide chemistry are just two of the options in which the thiol moieties within the GMP-SHs could be utilized. The present work has demonstrated GMP-SHs as a versatile platform technology that would enable the controlled delivery, presentation and release of factors, growth factors, or drugs. The thiol chemistries used to modify the particles are independent of microparticle crosslinking chemistry and degradation, demonstrating the platform’s strength in a variety of applications.

## Conclusion

GMP-SHs were created and their ability to serve as a modular and novel delivery platform for cells and small molecules was demonstrated. The microparticle physicochemical properties were assessed compared to a GMP control and the presence of thiols was not found to significantly impact microparticle swelling behavior. This demonstrates the promise of GMP-SHs as an additive to carry cells or bioactive factors in injectable and extrusion-based 3D printing applications. The crosslinking density of the microparticles was decreased for the GMP-SH 20 group compared to GMP-SH 80 and GMP controls, yet they exhibited no more than 10% mass loss throughout the accelerated degradation study. This is comparable to the degradation observed for the GMP and GMP-SH 80 groups, demonstrating the stability of the microparticles even in conditions designed to mimic the wound healing environment. The microparticle leachables were shown to be cytocompatible and the thiolated particles were furthermore shown to be a robust and novel substrate for MSC attachment and activity. Finally, the thiolated particles were proven to be a modular platform in their ability to be covalently modified with a maleimide-bearing fluorescent dye and an N-cadherin mimic peptide post-fabrication. The microparticle group bearing the lowest thiol content, GMP-SH 20, was capable of bearing as much of the loaded fluorophore as the GMP-SH 80 group, indicating the platform’s strength in the ability to simultaneously minimize thiol content while maximizing the hydrophobic fluorophore-loading. Moreover, the GMP-SH 20 and 80 platforms demonstrate promise in their ability to serve as a platform for dosed bioactive peptide delivery as seen in the differed loading of the GMP-SH 20 and 80 groups when reacted with the N-cadherin mimic peptide.

The ability to deliver cells and/or factors within biomaterials-based tissue engineering systems is crucial. Microparticles enable delivery of these components in a modular fashion where the bioactive cues and cells can be delivered in simple compositional ratios instead of changing the entirety of the bulk scaffold material. This modularity facilitates high-throughput analysis that shows promise in a variety of tissue engineering and drug delivery applications. The novel GMP-SH 20 and 80 microparticles generated in this work were demonstrated to possess the same cytocompatibility as GMPs, while facilitating the covalent, post-fabrication modification of the particles to carry small molecules. This innovative platform shows promise for a variety of biomaterial applications within the fields of tissue engineering and drug delivery as it can serve as a single platform for cell or factor delivery and shows promise for the controlled presentation of bioactive factors.

## Supplementary data

Supplementary data are available at *REGBIO* online.

*Conflict of interest statement*. The authors declare no conflicts of interest. 

## Supplementary Material

rbab012_Supplementary_Data
