# Supplementary material for: Development of a modular, biocompatible thiolated gelatin microparticle platform for drug delivery and tissue engineering applications
Source: Regen Biomater. 2021 Feb 28;8(3):rbab012. doi: 10.1093/rb/rbab012 (PMC8240604; doi:10.1093/rb/rbab012)
Supplement: rbab012_Supplementary_Data [file rbab012_supplementary_data.zip › rbab012_Supplementary_Data/Supplementary Information 2.7.21 Compressed.docx]

**Supplementary Information**

Size distribution of microparticles swollen in PBS as measured via image analysis is given below (**Supplementary Figure 1**). A full statistical letter report where different letters denote statistical significance (p < 0.05) for the degradation data is provided (**Supplemental Figure 2**). Additionally, the dsDNA data from the L929 leachables biocompatibility assay is provided demonstrating no statistical significance between groups (p < 0.05) (**Supplementary Figure 3**). The MALDI TOF spectra (**Supplementary Figure 4**) and the ^1^H-NMR spectra for the maleimide bearing N-cadherin peptide (**Supplementary Figure 5**) are provided.


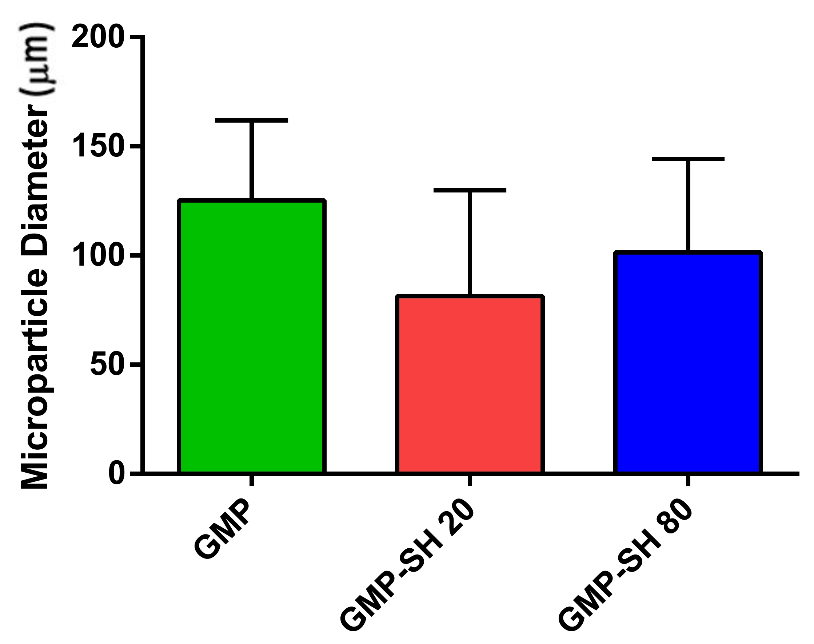


**Supplementary Figure 1**. **Size Distribution of the Gelatin and Thiolated Gelatin Microparticles**. Microparticles were swollen in PBS and imaged via light microscopy. The microparticles were then measured and the mean microparticle size is presented ± standard deviation (n = 10 per group). No statistical significance was detected between groups (p < 0.05).


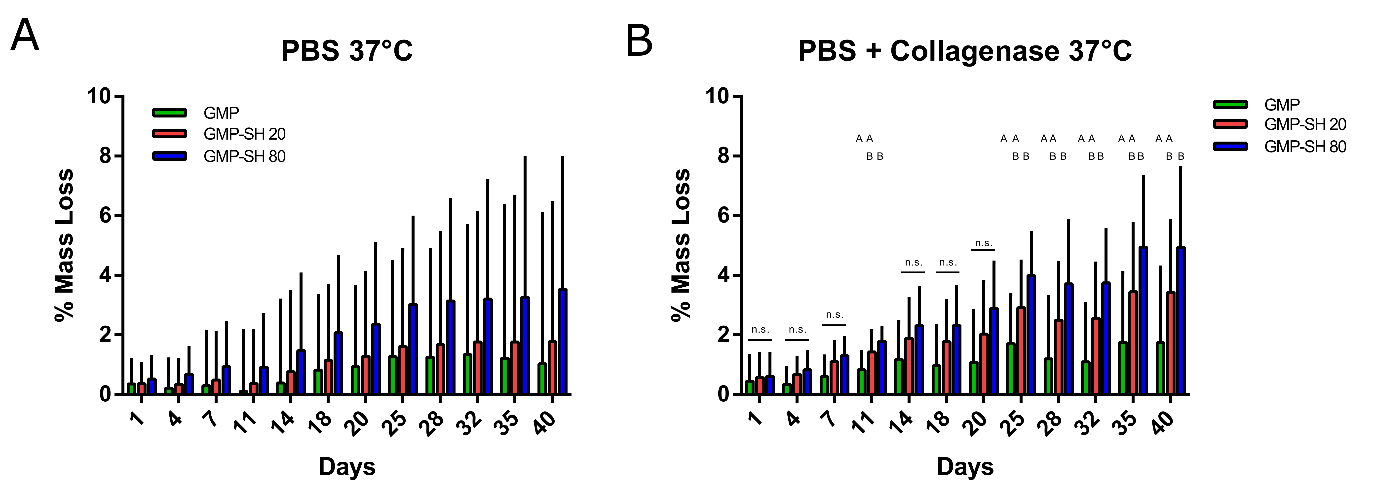


**Supplementary Figure 2**. **Letter Report for the In Vitro Degradation of Gelatin and Thiolated Gelatin Microparticles**. Degradation for each microparticle group is reported as percent (%) mass loss over a 40-day degradation study in **(A)** PBS and **(B)** PBS + Collagenase (800 ng/mL). Data is reported as means ± standard deviation for n=4 replicates per group. Shared letters denote no statistical significance for p < 0.05 within each day and degradation medium. No statistical significance (n.s.) between groups was detected in PBS conditions **(A)** within each timepoint and in collagenase-containing PBS **(B)** for the days noted above.


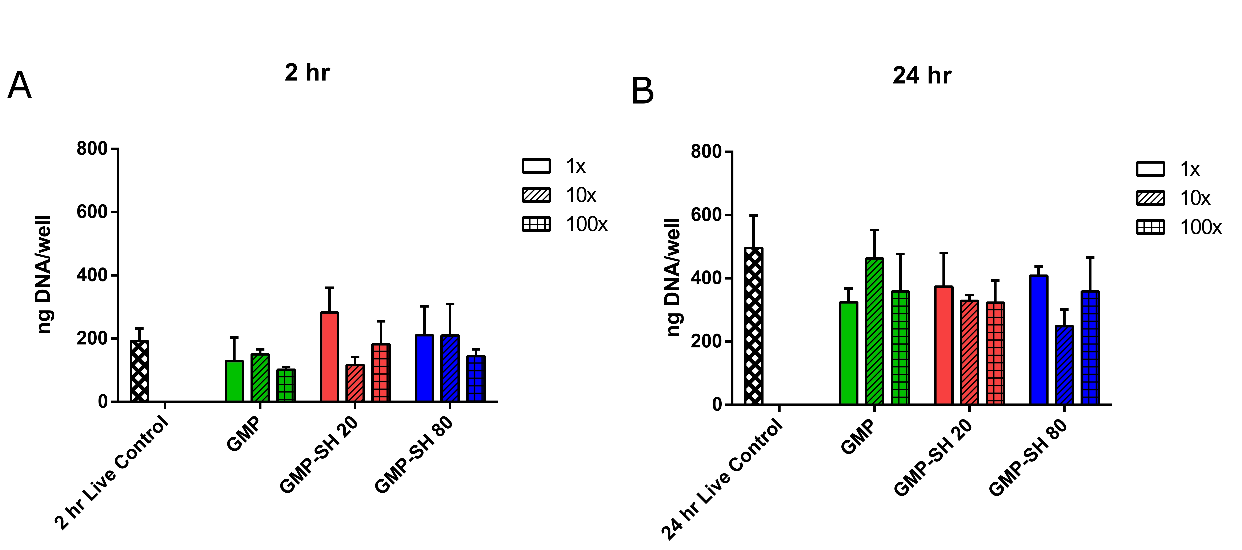


**Supplementary Figure 3**. **PicoGreen DNA Quantification of L929 Fibroblasts Treated with Microparticle Leachables**. Following metabolic activity assessment via WST1 assay of the L929 fibroblasts cells after 2 and 24 hr leachables exposure, the cells were lysed and their DNA quantified via PicoGreen assay. Data is reported as means ± standard deviation for n=3 replicates per group. No statistical significance was found between groups within the same timepoint (p < 0.05).


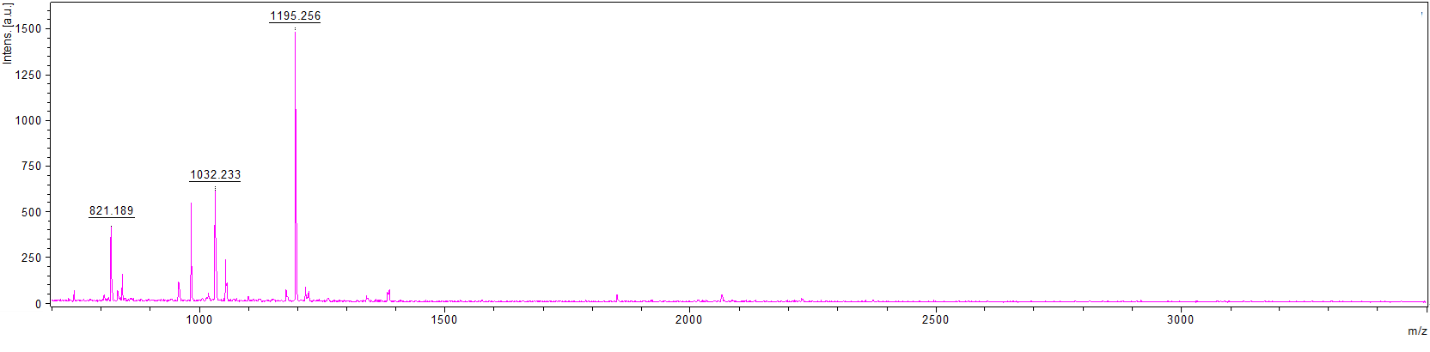

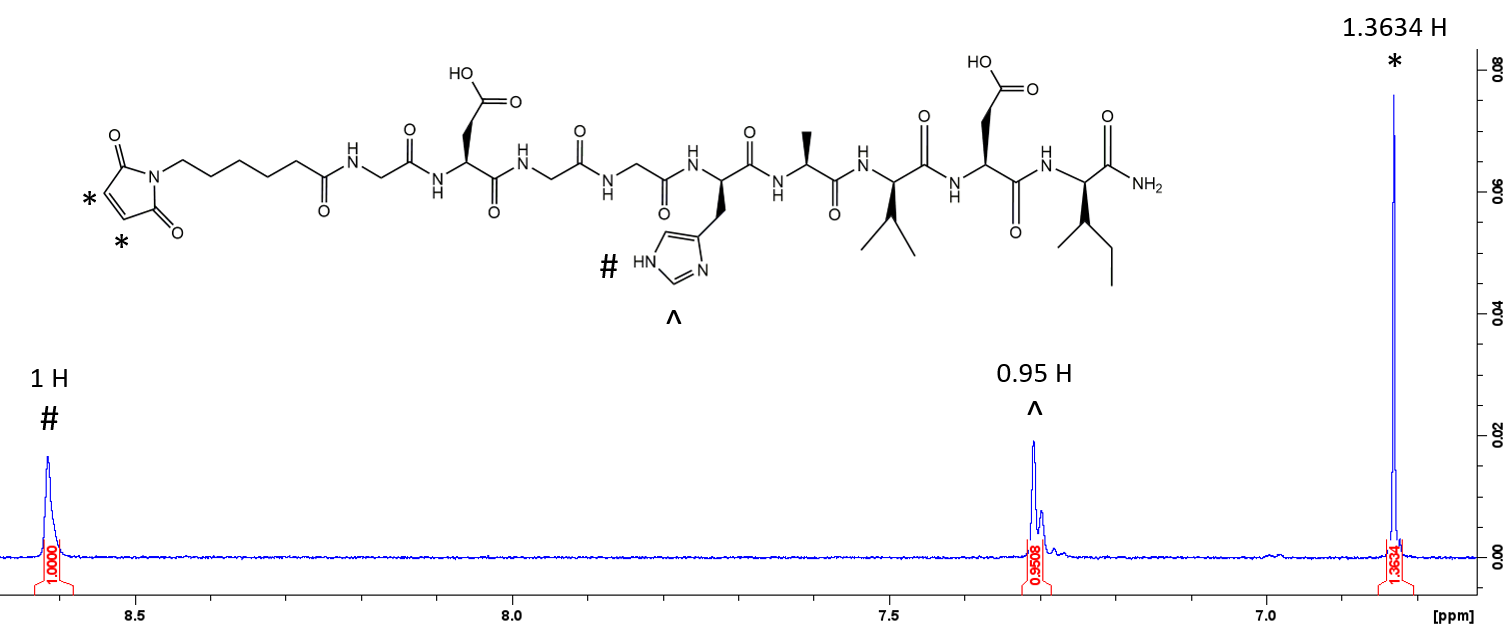


**Supplementary Figure 4**. **MALDI TOF for N-cadherin Mimic Peptide**. Following peptide synthesis and purification, the peptide was confirmed via MALDI TOF in a CHCA matrix dissolved in acetonitrile, water, and TFA. The peak at 1032.233 represents the maleimide bearing peptide sequence and the peak at 821.189 represents the peptide without the maleimide group. The peak at 1195.256 represents the peptide bound with acetonitrile and TFA.

**Supplementary Figure 5**. **^1^H-NMR spectra for N-cadherin Mimic Peptide**. Following peptide synthesis and purification, the peptide sequence and maleimide modification was confirmed via ^1^H-NMR. The peaks at 8.6 and 7.3 were confirmed as the protons on the histidine as indicated above with the peak at 8.6 used as the reference for all calculations. Integration of the protons corresponding to the maleimide at 6.8 was performed to determine the extent of maleimide addition to the peptide, which was calculated to be 68%.
