# Supplementary material for: Development of a modular, biocompatible thiolated gelatin microparticle platform for drug delivery and tissue engineering applications
Source: Regen Biomater. 2021 Feb 28;8(3):rbab012. doi: 10.1093/rb/rbab012 (PMC8240604; doi:10.1093/rb/rbab012)
Supplement: rbab012_Supplementary_Data [file Response to Reviewers Regenerative Biomaterials 2.8.21 330 2.pdf › rbab012_Supplementary_Data/Response to Reviewers Regenerative Biomaterials 2.8.21 330 2.pdf]

Development of a Modular, Biocompatible Thiolated Gelatin Microparticle Platform for Drug Delivery and Tissue Engineering Applications

Manuscript ID: RB-2021-002

We thank the reviewers for the constructive review of our manuscript and the insightful comments and suggestions. The modified manuscript, based on reviewer feedback, is attached with changes highlighted in yellow. Please find below the various comments of the reviewers in bold type followed by the author responses and the new text in the manuscript, highlighted in yellow.

Reviewer: 1

The submitted manuscript addresses a very important need in the field of regenerative biomaterials, namely the need to improve the modularity of scaffolds. Microparticles have been widely used in regenerative medicine and drug delivery and they are suggested by the authors as the vehicle to improve the modularity of polymeric scaffolds. The use of thiolated gelatin as such vehicle appears as a simple solution that can provide to future researchers great flexibility in the creation of modular scaffolds while maintaining the majority of the critical properties that scaffolds have pre-modification. The authors provide a complete set of experiments to prove the flexibility of the proposed methodology, including thiolation levels, thorough physicochemical characterization, cytocompatibility, ability to support MSC cultures, and demonstration of post-modification with a fluorophore and a relevant to regenerative medicine peptide of significant molecular weight. The experiments are very supportive of the proposed methodology and are properly conducted including all necessary controls. The description of the experimental methods is detailed.

Thank you so much for this very helpful and constructive feedback on our work.

1. The authors in the introduction and early in the Results and Discussion section refer to a "variety of tissue engineering applications that can benefit from the proposed methodology". Although I can personally imagine such examples, the manuscript will become stronger with the specific inclusion of few of such examples by name.

We agree. To provide further context of some of the possible tissue engineering applications, the following sections in the Introduction and Results and Discussion have been edited to incorporate some of these examples.

The modular platform was also examined to determine its capability of serving as a cell or factor delivery vehicle for use in future drug delivery or tissue engineering applications such as osteochondral tissue engineering, wound healing, and more.

The ability to covalently modify GMPs post-fabrication enables their use as a modular building block in a variety of tissue engineering applications such as bone and cartilage tissue engineering, wound healing applications, and more.

2. An estimate of the size distribution, before and after the thiolation, using the images of the microparticles could be helpful to confirm what can be seen in Figure 3, that the

thiolation appears to not affect the size of the microparticles.

We agree, thank you for this very helpful suggestion. Microparticle size distribution was calculated via image analysis of the microparticles after swelling in PBS and imaging via light microscopy. The following sections were added into the manuscript to outline these findings and the figure added to the Supplemental Information.

Microparticle size distribution was determined via image-based analysis of particles swollen in PBS with average particle sizes determined to be 125.2 ± 34.8, 81.4 ± 46.1, and 101.4 ± 40.6 µm for the GMP, GMP-SH 20, and GMP-SH 80 groups, respectively. The size distribution of the microparticles is provided in **Supplementary Figure 1** and demonstrates no significant difference in size between groups.

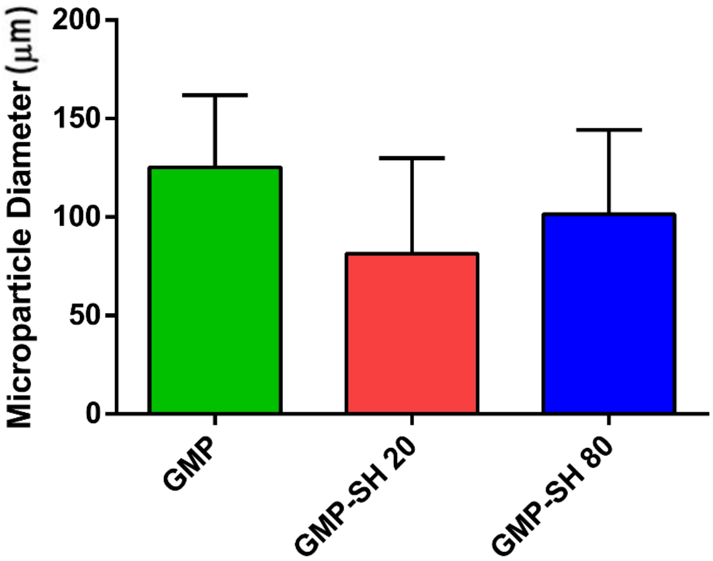

Supplementary Figure 1. **Size Distribution of the Gelatin and Thiolated Gelatin Microparticles**. Microparticles were swollen in PBS and imaged via light microscopy. The microparticles were then measured and the mean microparticle size is presented ± standard deviation (n = 10 per group). No statistical significance was detected between groups ($p < 0.05$).

3-5. In Figure 4, parts C and D (mostly C) a better choice of colors can assist in discriminating the error bars.

In many figures a line on top of graphs appears that denotes no statistical significance. I find this choice interesting and a potential source of confusion. It is probably adequate to just include the absence of statistical difference in the Figure caption.

In figure 5 it becomes apparent that the earlier choice of colors continues to reflect the different thiolation level groups throughout. I think a more dramatic choice of colors

would enhance the visual effect and make the figures more appealing (in conjunction with my comment #3).

Thank you for these comments. We agree that more dramatic color choices between groups will aid in the ability to discriminate between groups and will aid in our communication of the results. We appreciate the comments as well recommending the removal of “n.s.” from the graphs themselves and simply denoting the lack of significance in the figure captions. The following figures and figure captions have been changed to incorporate these recommendations.

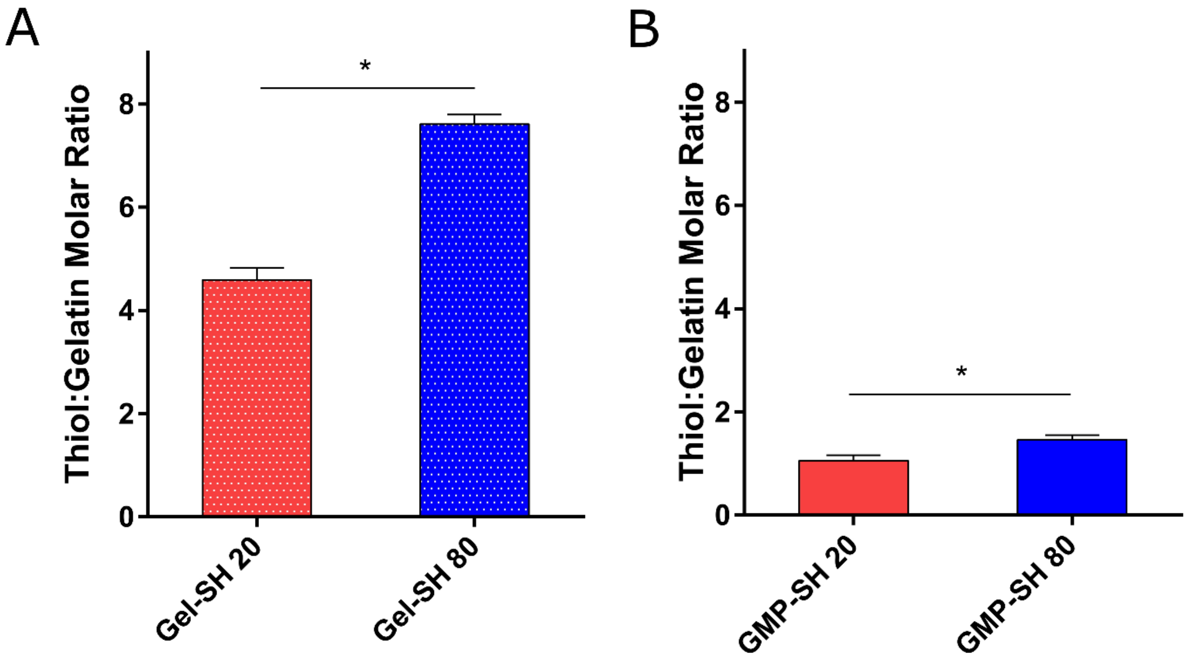

Figure 2. **Thiol Content for the Thiolated Gelatin and Thiolated Gelatin Microparticle Groups. (A)** Thiolated gelatin (Gel-SH 20 and 80) and **(B)** thiolated gelatin microparticle groups (GMP-SH 20 and 80) were assessed for thiol content via a modified Ellman’s assay using a cysteine standard curve. Data is reported as means ± standard deviation for n=3 replicates per group. * Indicates significant differences between groups ($p < 0.05$).

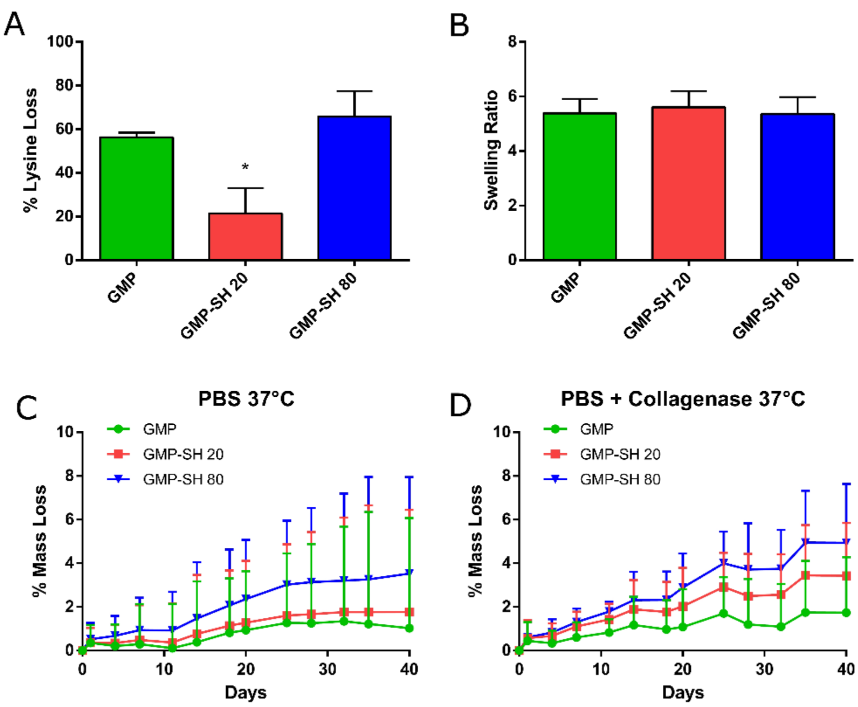

Figure 4. **Physicochemical Characterization of the Thiolated Gelatin Microparticle Groups. (A)** Crosslinking density was determined as % lysine loss measured via TNBSA assay. Data is reported as means ± standard deviation for n=3 replicates per group. *Indicates significant difference ($p < 0.05$). **(B)** Swelling ratio of the thiolated particle groups compared to GMP control. Data is reported as means ± standard deviation for n=5 replicates per group. No statistical significance was detected ($p < 0.05$). **(C)** Degradation of the thiolated and control gelatin microparticle groups in PBS at 37°C. Data is reported as means + standard deviation for n=4 replicates per group**. (D)** Degradation of the thiolated and control gelatin microparticle groups in collagenase-containing medium at 37°C. Data is reported as means + standard deviation. N=4 replicates per group.

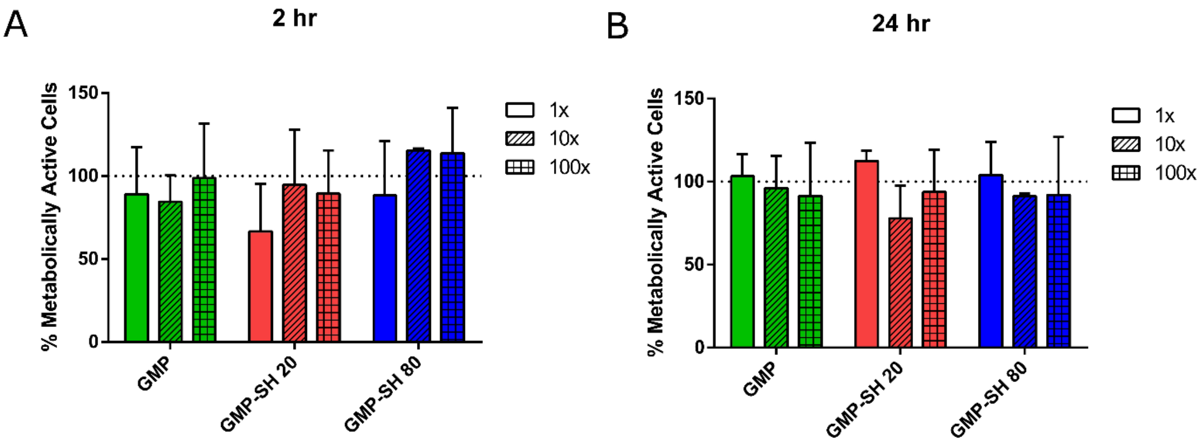

Figure 5. **WST1 Metabolic Assay of L929 Fibroblasts Treated with Microparticle Leachables**. Metabolic activity of the cells was assessed via WST1 assay at **(A)** 2 and **(B)** 24 hr following leachables exposure. Data is reported as means ± standard deviation for n=3 replicates per group. No statistical significance was found between groups within the same timepoint ($p < 0.05$).

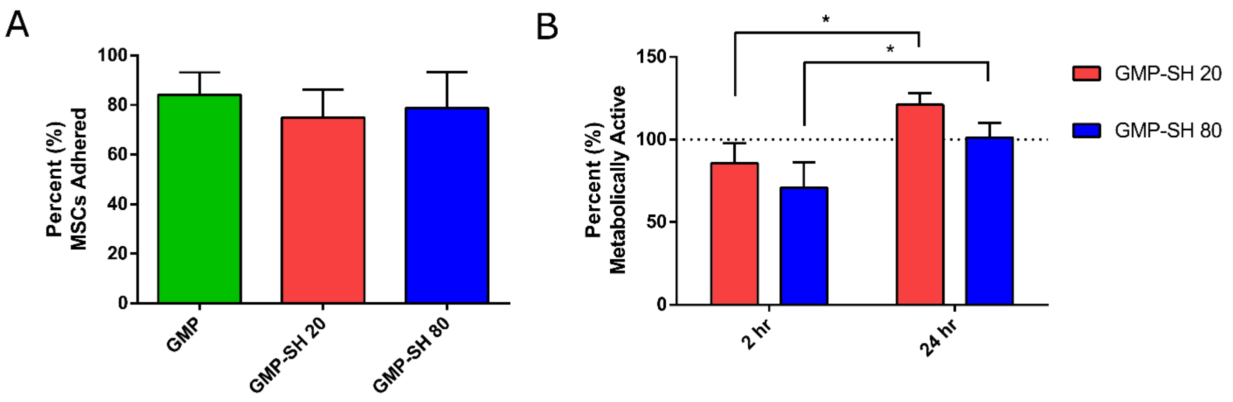

Figure 7. Quantification of MSC Adherence to Gelatin and Thiolated Gelatin Microparticles and Adhered MSC Metabolic Activity. MSC-microparticle aggregates were analyzed via Picogreen assay to determine **(A)** percent (%) adherence to the microparticles. Data is reported as means ± standard deviation for n=3 replicates per group. No significant difference was detected between groups ($p < 0.05$)**.** **(B)** The aggregates were cultured in rotational culture and the metabolic activity of the adhered MSCs was examined at 2 and 24 hr via WST1 assay. Data is reported as means ± standard deviation for n=3 replicates per group. The dashed line indicates the activity of MSCs adhered to GMP controls. * Indicates significant differences within each group between the activity observed at 2 hr compared to 24 hr ($p < 0.05$).

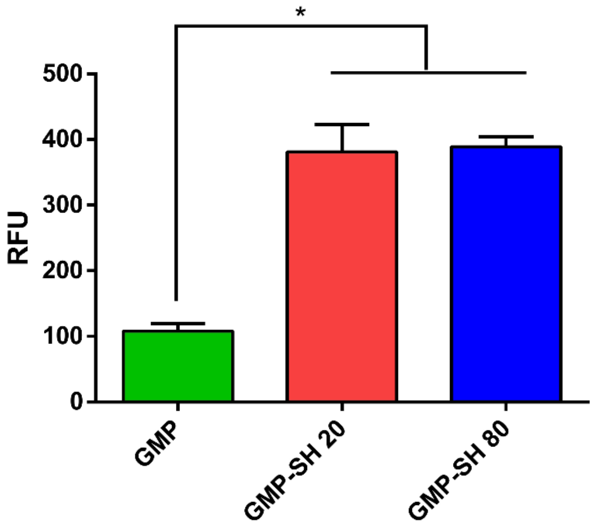

Figure 8. Fluorescence of Microparticle Groups Incubated with Alexa Fluor-Maleimide (AF-Mal) 488. Fluorescence of the microparticles was analyzed and normalized to gelatin content. Data is reported as means ± standard deviation for n=3 replicates per group. * Indicates significant differences between groups ($p < 0.05$).

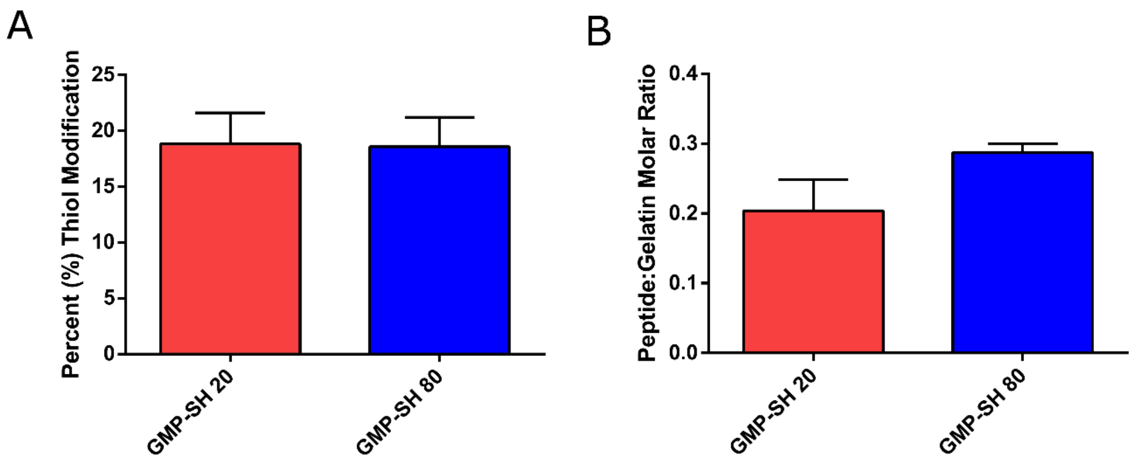

Figure 10. **Modification of the Thiolated Microparticles with an N-Cadherin Mimic Peptide**. The percent of thiol modification **(A)** with the maleimide-bearing peptide was determined via Ellman's assay. Data is reported as means ± standard deviation for n=4 replicates per group. No statistical significance was found between groups ($p < 0.05$). The peptide molar modification ratio **(B)** was then calculated using the known molar content of the GMP-SH 20 and 80 groups. Data is reported as means ± standard deviation for n=3 replicates per group. No statistical significance was found between groups ($p < 0.05$).

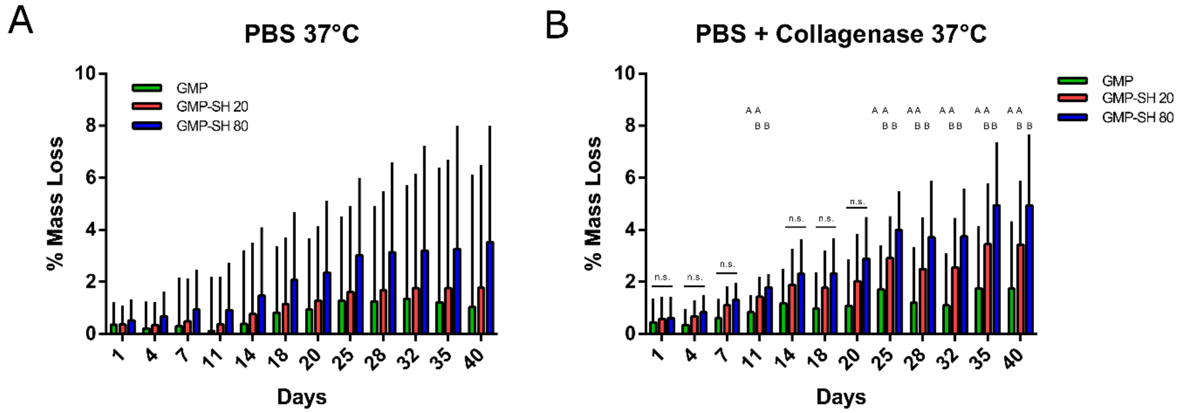

Supplementary Figure 2. **Letter Report for the In Vitro Degradation of Gelatin and Thiolated Gelatin Microparticles**. Degradation for each microparticle group is reported as percent (%) mass loss over a 40-day degradation study in **(A)** PBS and **(B)** PBS + Collagenase (800 ng/mL). Data is reported as means ± standard deviation for n=4 replicates per group. Shared letters denote no statistical significance for $p < 0.05$ within each day and degradation medium. No statistical significance (n.s.) between groups was detected in PBS conditions **(A)** within each timepoint and in collagenase-containing PBS **(B)** for the days noted above.

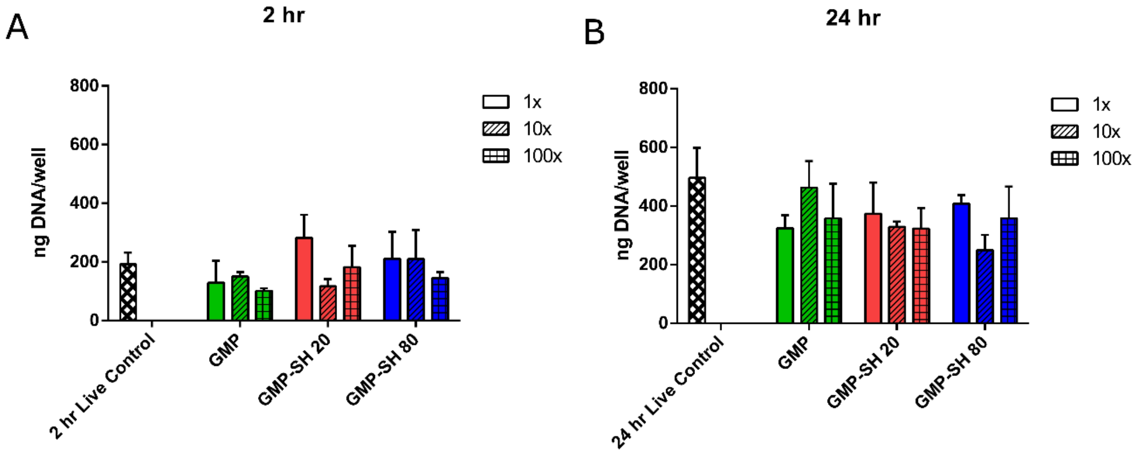

Supplementary Figure 3. **PicoGreen DNA Quantification of L929 Fibroblasts Treated with Microparticle Leachables**. Following metabolic activity assessment via WST1 assay of the L929 fibroblasts cells after 2 and 24 hr leachables exposure, the cells were lysed and their DNA quantified via PicoGreen assay. Data is reported as means ± standard deviation for n=3 replicates per group. No statistical significance was found between groups within the same timepoint ($p < 0.05$).

Reviewer: 2

This report describes thiolated gelatin microparticles (GMP-SHs) with the ability to be functionalized post-fabrication through addition to the thiol groups, thereby increasing the modularity of GMP systems useful in drug delivery and in tissue engineering. GMPs with a “high” (GMP-SH-80) and “low” (GMP-SH-20) thiol concentration were evaluated and compared to a GMP control (i.e., no thiolation). The characterization of these microparticles was robust and thorough. Overall, the GMP-SHs, despite differences in crosslinking density, maintained similar swelling and were rather stable in even accelerated degradation conditions. GMP-SHs leachates were cytocompatible and were suitable substrates for MSCs. An Alexa-Fluor maleimide dye (AF-MAL 488) was conjugated to the GMP-SHs via a thiol-maleimide reaction, at similar levels for GMP-SH-80 and GMP-SH-20. Thus, these GMP-SHs have interesting potential for drug delivery and tissue engineering.

Thank you very much for the helpful and constructive feedback on our work.

1. The authors note that "Particles between 50-100 microns while dry were used for all studies…were isolated via sieving." Was the size confirmed? (e.g. SEM/Image J analysis?)

Thank you very much for this comment. We agree that characterizing microparticle size strengthens the work. To this end, we performed image analysis on the microparticles while swollen in PBS and have reported their size distribution, as well as included a new figure in the supplemental information.

Microparticle size distribution was determined via image-based analysis of particles swollen in PBS with average particle sizes determined to be 125.2 ± 34.8, 81.4 ± 46.1, and 101.4 ± 40.6 µm for the GMP, GMP-SH 20, and GMP-SH 80 groups respectively. The size distribution of the microparticles is provided in **Supplementary Figure 1** and demonstrates no significant difference in size between groups.

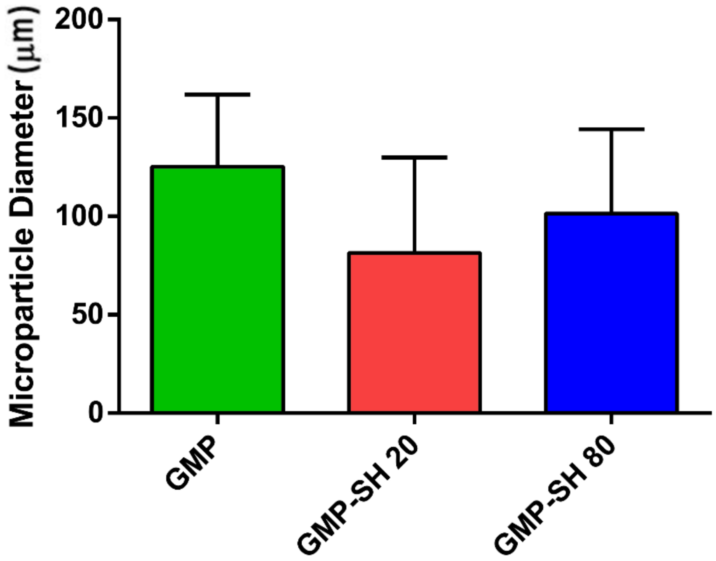

Supplementary Figure 1. Size Distribution of the Gelatin and Thiolated Gelatin Microparticles. Microparticles were swollen in PBS and imaged via light microscopy. The microparticles were then measured and the mean microparticle size is presented ± standard deviation (n = 10 per group). No statistical significance was detected between groups ($p < 0.05$).

2. Please consider re-wording this sentence for clarity: (Conclusion) "The crosslinking density of the microparticles was impacted for the GMP-SH 20 group, yet they maintained stability throughout the degradation study."

Thank you for this helpful comment. The following changes have been made in the body of the manuscript to increase clarity.

The crosslinking density of the microparticles was decreased for the GMP-SH 20 group compared to GMP-SH 80 and GMP controls, yet they exhibited no more than 10% mass loss throughout the accelerated degradation study. This is comparable to the degradation observed for the GMP and GMP-SH 80 groups, demonstrating the stability of the microparticles even in conditions designed to mimic the wound healing environment.

3. Perhaps the authors can comment on if the GMP-SHs may be "too stable" for some applications and how that might be addressed.

Thank you for this helpful comment. The following information has been incorporated into the Results and Discussion to provide some information for those who may be interested in a more accelerated degradation profile of the microparticles.

Previously, our group has demonstrated the ability to tune degradation for the purposes of growth factor release by varying the glutaraldehyde crosslinker concentration during microparticle fabrication[6,17,52]. A similar schema could be employed of reducing the glutaraldehyde concentration from 40 to 10 mM in the future if a more accelerated degradation profile is required[6,15,17,52].
